# Wireless monitoring of respiration with EEG reveals relationships between respiration, behavior, and brain activity in freely moving mice

**DOI:** 10.1152/jn.00330.2023

**Published:** 2024-05-29

**Authors:** Debanjan Dasgupta, Deborah Schneider-Luftman, Andreas T. Schaefer, Julia J. Harris

**Affiliations:** ^1^Sensory Circuits and Neurotechnology Laboratory, The Francis Crick Institute, London, United Kingdom; ^2^Department of Neuroscience, Physiology & Pharmacology, University College London, London, United Kingdom; ^3^UK Dementia Research Institute, University College London, London, United Kingdom; ^4^UCL Sainsbury Wellcome Centre for Neural Circuits and Behaviour, London, United Kingdom; ^5^Neural Circuit Dynamics Laboratory, Department of Biological Sciences and Bioengineering, Indian Institute of Technology, Kanpur, India

**Keywords:** EEG, exploration, respiration, sleep

## Abstract

Active sampling in the olfactory domain is a fundamental aspect of mouse behavior, and there is increasing evidence that respiration-entrained neural activity outside of the olfactory system sets an important global brain rhythm. It is therefore crucial to accurately measure breathing during natural behaviors. We develop a new approach to do this in freely moving animals, by implanting a telemetry-based pressure sensor into the right jugular vein, which allows for wireless monitoring of thoracic pressure. After verifying this technique against standard head-fixed respiration measurements, we combined it with EEG and EMG recording and used evolving partial coherence analysis to investigate the relationship between respiration and brain activity across a range of experiments in which the mice could move freely. During voluntary exploration of odors and objects, we found that the association between respiration and cortical activity in the delta and theta frequency range decreased, whereas the association between respiration and cortical activity in the alpha range increased. During sleep, however, the presentation of an odor was able to cause a transient increase in sniffing without changing dominant sleep rhythms (delta and theta) in the cortex. Our data align with the emerging idea that the respiration rhythm could act as a synchronizing scaffold for specific brain rhythms during wakefulness and exploration, but suggest that respiratory changes are less able to impact brain activity during sleep. Combining wireless respiration monitoring with different types of brain recording across a variety of behaviors will further increase our understanding of the important links between active sampling, passive respiration, and neural activity.

**NEW & NOTEWORTHY** Animals can alter their respiration rate to actively sample their environment, and increasing evidence suggests that neurons across the brain align their firing to this changing rhythm. We developed a new approach to measure sniffing in freely moving mice while simultaneously recording brain activity, and uncovered how specific cortical rhythms changed their coherence with respiration rhythm during natural behaviors and across arousal states.

## Graphical abstract

**Figure:**
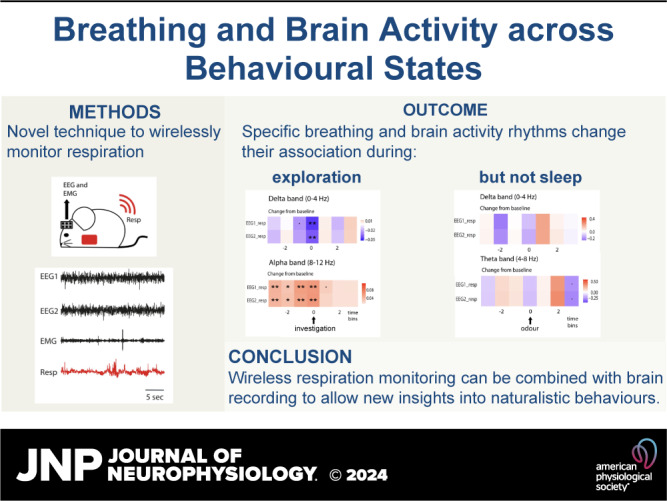

## INTRODUCTION

Active sampling plays a crucial role in sensory processing (1), particularly within the sense of olfaction (2–8). Olfactory sampling is governed by the respiration rhythm, and many mammals display a huge repertoire of “sniffing” behaviors that dynamically alter their respiration rate (6, 7, 9–11). In turn, respiration-entrained neuronal firing patterns are increasingly being observed across brain regions outside of the olfactory system, including the hippocampus (12–14), neocortex (15–17), and limbic system (18–20) (for review, see Ref. 21). Accumulating evidence has led to the hypothesis that breathing sets a global brain rhythm to actively coordinate neuronal communication across distant brain regions (22–24) that may contribute to learning and memory (25) and the expression of learned behavior (17). It is now being discovered that the alignment of different neural activity patterns to this respiration rhythm can change with the arousal state and behavior of the animal (26–31). During exploration, respiratory rhythms overlap in frequency with the hippocampal theta rhythm (32) and confusion may therefore arise in the absence of knowledge about the animal’s breathing. Accurately measuring sniffing is therefore critical not only in the fields of respiratory physiology and olfaction, but also in the context of animal behavior and neural processing more generally.

Over the years, different strategies have been developed to monitor respiration (33), including whole body plethysmography (34–37); monitoring chest distension using piezoelectric bands (38, 39); thermal imaging of nostrils (40); and monitoring changes in nasal air flow (38, 41–44), pressure (6, 45–47), or temperature (48–53). However, these methods all require specific recording conditions or tethering, making it difficult to measure respiration during natural behaviors or in combination with other recording techniques. Recently, an innovative approach was developed, which has the potential to overcome this challenge. Specifically, a wireless pressure sensor was threaded alongside the esophagus and implanted into the thoracic cavity, where it could detect thoracic pressure changes that directly reflect respiration (46). This approach was applied in awake, freely moving animals, allowing respiration to be monitored in a natural environment and during behavioral tasks.

In the current study, we present a new surgical approach to implant a wireless thoracic pressure sensor into the jugular vein, and verify the thoracic pressure signal against a head-fixed flow-sensor recording. Probe insertion into the jugular vein, rather than alongside the esophagus wall (46) simplified the postoperative care, with no requirement for a change to liquid-based diet. This surgical approach is thus more suitable for typical learning assays where water restriction is required for training. We then use this technique in conjunction with implanted electroencephalography (EEG) and electromyography (EMG) electrodes to measure brain activity in freely moving mice. We examined respiration pattern changes as mice voluntarily explored novel objects, novel odors, and food odors, and also across different arousal states, while simultaneously examining the relationship between respiration and cortical rhythms. Gaining insight into how respiration relates to brain activity and behavior will provide new opportunities to understand how animals process the world around them and interact with their environment.

## MATERIALS AND METHODS

### Implantation of Thoracic Pressure Sensor in Right Jugular Vein

All animal procedures performed in this study were regulated and approved by the Institutional Animal Welfare Ethical Review Panel and the UK government (Home Office) under license PA2F6DA12. All the surgeries and behavioral assays were performed on male C57/Bl6 mice.

**Figure 1.**
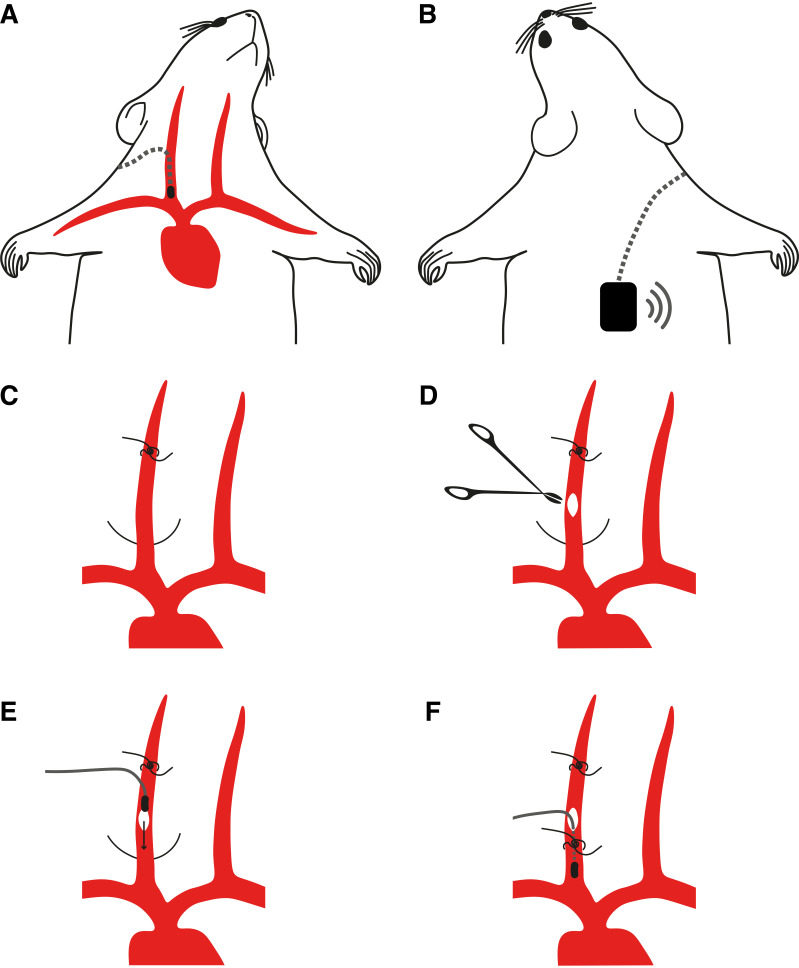
Jugular vein implantation of thoracic pressure sensor. *A*: schema showing the final location of the pressure sensor tip in the jugular vein and the subcutaneous location of the transmitter on the back (*B*). *C*–*F*: the critical steps of the surgery are tying the rostral knot on the right jugular vein (*C*); making the incision on the jugular vein (*D*); inserting the sensor tip through the incision toward the heart (*E*); and securing the sensor at the correct position for optimal signal (*F*).

Thoracic pressure sensors (Stellar implantable transmitter device, 10 × normal gain, E-430001-IMP-22, TSE systems, Germany) were implanted in the right jugular vein in mice. Animals 6–8 wk old were put in individual cages 2 days before surgery to ensure acclimatization and proper intake of drugs orally. On the day prior to the surgery, 0.2 mL of egg custard (Cow & Gate) + 0.2 mL oral Metacam suspension was given in addition to freely available food. On the day of surgery, the animal was weighed and anesthetized using fentanyl (0.05 mg/kg) + midazolam (5 mg/kg) + medetomidine (0.5 mg/kg), delivered intraperitoneally. Furthermore, for analgesia meloxicam (10 mg/kg) + buprenorphine (0.1 mg/kg) was administered subcutaneously. The animals were then placed on a heatpad (DC Temperature Controller, FHC, Bowdoin, ME) controlled by a rectally inserted temperature sensor. Body temperature was continuously monitored and maintained at 37 ± 0.5°C. The probes consisting of two parts, a transmitter (2 cm × 1 cm × 0.3 cm) and a catheter (5-cm long and ∼0.4-mm diameter), were implanted using aseptic surgery techniques. The skin on the right of the neck’s midline was shaved and disinfected with 25% (vol/vol) chlorhexidine. Next, a ∼2-cm skin incision was made and using blunt tools a subcutaneous tunnel was created underneath the right arm up to the back of the animal. Presterilized saline solution was used to irrigate the wound regularly. The transmitter was pushed through this tunnel up to the back of the animal (Fig. 1*B*) while keeping the sensor end out of the wound. Next, postisolation of the right jugular vein, a knot was tied on the dorsal-most part of the isolated section of the vein using nonsoluble surgical sutures (Fig. 1*C*). A small incision was made on the vein surface to insert the sensor tip of the catheter (Fig. 1*D*). During the process of insertion of the sensor tip (Fig. 1*E*), the pressure signal was continuously monitored to identify the best spot for placement. Upon reaching the best spot, a knot was firmly tied around the vein enclosing the catheter (Fig. 1*F*). The remaining suture thread from the dorsal knot was also used to make a knot around the catheter for extra stability of the placed sensor. Finally, the wound was closed using 6-0 silk suture and a reverse-cutting needle. The animal was recovered in a heated chamber after injecting 1.2 mg/kg naloxone + 0.5 mg/kg flumazenil + 2.5 mg/kg atipamezole (ip). Sterile saline (0.2 mL) was injected subcutaneously for faster recovery. The animals were monitored and their bodyweights were recorded regularly at least for the next 10 days postsurgery.

For the head-fixed recordings, a subset of animals also underwent a head-fixation implant attachment in the same surgery. Briefly, a custom-made head-fixation implant was glued to the skull with a medical grade superglue (Vetbond, 3M, Maplewood, MN). Furthermore, dental cement (Palladur, Heraeus Kulzer GmbH, Hanau, Germany) was applied around the base of the implant to strengthen the fixation.

Postsurgery, the animals were allowed at least a week to recover from the surgery and to get back to their presurgery body weights before being used for experiments.

### EEG and EMG Electrode Implantation

For the EEG/EMG exploration and sleep experiments, mice underwent a second surgery at least 1 wk after implantation of the telemetry device. Mice were anesthetized with isoflurane and injected subcutaneously with meloxicam (2 mg/kg body wt) for analgesia. After positioning in a stereotaxic frame (Kopf Instruments), mice were implanted with four miniature screw electrodes (from bregma: AP +1.5 and ML +1.5 (ground); AP +1.5 and ML −1.5 (common reference); AP −1.5 and ML −1.5 (EEG 1); AP −1.5 and ML +1.5 (EEG 2) and two EMG electrodes (inserted into neck musculature). These electrodes were each connected, via an insulated wire, to a different gold pin of a EEG/EMG headstage. The EEG/EMG headstage was affixed to the skull using dental adhesive resin cement (Super-Bond C&B). Mice were allowed to recover for a further week before participating in head-fixed and then freely moving behavior experiments.

### Data Acquisition of the Telemetric Thoracic Pressure Signal

A commercial telemetry system associated with the probes (TSE systems) was used for the wireless recording of the thoracic pressure signals in awake animals. The signal from the probe’s transmitter was sensed by the antenna of the telemetry system output that was connected to DAQ (CED Micro1401 with ADC12 expansion, Cambridge Electronic Design Limited, UK) and controlled by spike2 (Cambridge Electronic Design Limited, UK) on a computer. The signal was sampled at 1 kHz by the sensor and digitized at 10 kHz for the experiments on head-fixed animals (to synchronize with the flow sensor recording) and downsampled to 400 Hz for the experiments involving freely moving animals (to synchronize with the EEG/EMG recording).

Because the implanted probe has a fixed battery life, we could not acquire the thoracic pressure signal continuously. We therefore acquired in 0.5–2 min bursts, with onset timed according to each experiment (before object introduction in the exploration experiment, and after online detection of non-rapid eye movement (NREM) sleep in the sleep experiments).

### Data Acquisition of the Nasal Flow Sensor Signal

A mass flow sensor (FBAM200DU, SensorTechnics) was used to measure the flow change in front of the nostril thus generating a continuous respiration signal as described previously (43). The signal was digitized at 10 kHz simultaneously with the thoracic pressure signal using the same CED Micro1401 DAQ.

### Head-Fixed Experiments

The animals were placed in a custom-made head-fixation apparatus attached to a treadmill. The flow sensor was placed in front of the nostrils. Following a brief period of habituation (∼15 min), signals from the flow sensor and the thoracic sensor were recorded simultaneously, with acquisition as described earlier.

### Data Acquisition of the EEG/EMG Signals

EEG and EMG signals were recorded using the Pinnacle 3-channel tethered system (8200-K1-SL; Pinnacle Technology Inc.). Signals were filtered by the preamplifier (high pass above 0.5 Hz for EEG and above 10 Hz for EMG) and then recorded at a sample rate of 400 Hz in Spike2, via the same CED Micro1401 DAQ.

### Behavioral Experiments

During the behavioral sessions, mice were placed on the floor of a circular open-topped enclosure (50 cm in diameter) and video was recorded using a Raspberry Pi camera mounted on the ceiling (∼1.5 m above the enclosure). The camera sent out TTL pulses on a frame-by-frame basis (25 Hz) to the DAQ, which synchronized the respiration recording with the video recording. Formal animal tracking of head, center, and tail coordinates was performed offline using Ethovision XT software (Noldus). This allowed us to define exploratory approach and retraction times, and instantaneous velocity for all the frames of each trial. The following four types of behavioral experiment were performed, while video, respiration signal, and EEG/EMG (in a subset of mice) were recorded:

1)  Open arena exploration: The mouse was gently placed in the arena and allowed to explore freely for approximately 5 min.
2)  Novel object: Multiple objects (rubber duck, water bottle, nail varnish bottle, empty food hopper, black bottle, muffin toy) were placed in the arena one at a time, far away from the mouse, who was then allowed to approach and explore the object freely for approximately 5 min. Each object was only presented once to each mouse.
3)  Novel odor: A glass Petri dish containing a tissue piece impregnated with 2 mL of pure odor (ethyl butyrate, 2-hexanone, isopentyl acetate, or eucalyptol) was placed in the arena, far away from the mouse. The mouse was then allowed to approach and explore the Petri dish freely for approximately 5 min. Each odor was only presented once to each mouse during awake behavior (but for a second time during sleep, see below section, *Sleep Experiments*).
4)  Inaccessible food: A food pellet was placed in a meshed container, which was then placed in the arena, far away from the mouse. The mouse was then allowed to approach and explore the container freely for approximately 5 min. As these food pellets and their smell were already familiar to the mice, these stimuli were presented in 3–4 trials for each mouse.

### Sleep Experiments

EEG/EMG and respiration signals were recorded as the animal rested in its home cage, during the first half of the light phase. EEG signals were monitored online, to assess the arousal state of the animal in real time. When the animal entered NREM sleep, we waited for approximately 10 s before turning on the telemetry respiration sensor for 1 min. If the animal was still in NREM approximately 10 s after that, we carefully placed a Petri dish containing a tissue piece impregnated with 2 mL of pure odor (ethyl butyrate, 2-hexanone, isopentyl acetate, or eucalyptol; as in the awake behavior experiments, meaning that this was the second presentation of each odor), into the cage (as we did not see any consistent difference between odors, data were pooled across all four odors). Trials where the placement itself woke the animal up were excluded from analysis. The Petri dish was left in position in the cage until sniff monitoring for that trial ended (1 min). Trials were separated by at least 5 min.

### Data Analysis—Respiratory Signals

The data were analyzed using custom scripts written in MATLAB R2019 (MathWorks).

#### Frequency estimate.

The raw data were standardized and detrended. Next, 1 s of data were passed through an autocorrelation routine. The corresponding time of the first peak was estimated and its reciprocal was used to estimate the dominant frequency for that 1-s period. This was repeated using a rolling window of 1 s with a shift of 50 ms between adjacent windows.

For the freely moving experiment with novel cues, we noted the frames of start and end of each exploration bout and included 3 s preceding and following each exploration bout to make up an individual trial. We followed the same method for estimating frequency for all the trials thus extracted. Events with baseline time less than 3 s were discarded. All trials were aligned to the start of exploration. We calculated the average baseline frequency from the first 500 ms of the trial (this typically included ∼5 respiration cycles) and the average exploration frequency from the first 1 s of object exploration (Fig. 5, *M*–*O*). We then subtracted the baseline frequency from the entire trial to plot change in frequency, and this was averaged over trials and across mice (Fig. 5, *J*–*L*).

Frequency error calculation (Head-fixed): The frequency estimation for a 1-s period was estimated from both the flow sensor data and the thoracic sensor data. Next, the relative error for that 1 s was calculated as

$$\text{Relative error}=\frac{{\text{Freq}}^{\text{thoracic}}-{\;\text{Freq}}^{\text{flow}}}{{\text{Freq}}^{\text{flow}}}$$

The relative error is then averaged over the entire trial period to get an average relative error for the specific trial. The mean and standard deviation is estimated from all the trials from all the animals.

#### Inhalation peak detection.

The peak of inhalation was detected as the trough of every sniff cycle in the flow sensor signal and the simultaneously recorded thoracic sensor signal. The peaks were detected using custom-written scripts in MATLAB R2019 (MathWorks). The peak detection was manually scrutinized for discrepancies. To compare peak times between the thoracic and the flow sensor, inhalation peaks were detected in the signals recorded from both the sensors. Next, we calculated the difference in time between the two near simultaneous occurring peaks. Since the pressure change in the thoracic cavity happens slightly after any flow through the nostrils, it was assumed that the inhalation peak estimated from the flow sensor signal will precede that estimated from the thoracic sensor signal.

### Data Analysis—Behavior

Nose, center, and tail tracking were performed using Ethovision software. We estimated mouse position, head direction, and instantaneous velocity for each video frame of each trial. Mice were considered to have initiated an exploration bout as soon as their nose entered a 2 cm perimeter around the object/odor/food. Retraction time was defined as the next frame in which the mouse’s nose moved out of this perimeter space. In general, these definitions were not ambiguous, and classifying exploratory initiation and retraction times either automatically (using Ethovision) or manually (by scrolling frame-by-frame through the video) gave almost identical results.

### Data Analysis—Vigilance State Classification

Arousal states—NREM, rapid eye movement (REM), and wake—were automatically classified using sleep analysis software in Spike2, and then manually verified in 5-s epochs (as in Ref. 54). Wakefulness was defined as desynchronized, low-amplitude EEG and tonic EMG with bursts of movement. NREM sleep was defined as synchronized, high-amplitude EEG in the delta frequency range (1–4 Hz) and reduced EMG activity relative to wakefulness. REM sleep was defined when EEG had reduced delta power but prominent power in the theta range (4–10 Hz), and EMG showed an absence of muscle tone.

### Data Analysis—Association between EEG, EMG, and Novel Respiration Signals

#### Preprocessing.

Data recorded from the four physiology channels [EEG1, EEG2, EMG, and Respiration (Resp)] during each experiment were preprocessed before analysis, as follows. Exploration behavior markers derived from video recording were aligned with the timeline of the physiology recordings, using TTL time frame alignments. To save battery, the respiratory sensor was not activated throughout the whole experiment, and its recording was interrupted during some experiments. Thus, we removed all data points that had no Resp recording. We applied a Butterworth filter, with bandpass 0.5–120 Hz to the EEG channels. The resulting data set was then analyzed in four frequency bands of interest: delta (0.5–4 Hz), theta (4–8 Hz), alpha (8–12 Hz), and beta (12–30 Hz). This was achieved using band-specific band-pass filters.

#### Partial coherence estimates.

For the exploration experiments, we applied the slowly evolving locally stationary process (SEv-LSP) framework from Fiecas and Ombao to the preprocessed data (55). The four channels [EEG1, EEG2, EMG, Resp] recorded during each experiment were treated as a nonstationary four-dimensional time series. The four-dimensional time series were cut into small epochs (0.5–2 s long) which were treated as “locally stationary.” The choice of epoch length was chosen differently for each frequency band, to ensure that a single bin always contained approximately five full cycles: delta band: 2.22 s; theta band: 0.83 s; alpha band: 0.5 s; beta band: = 0.25 s.

For each experiment, and for each time epoch, the spectral power matrix was estimated using Thomson’s multitaper estimate (56) with *K* = *p* × 1.5 = 6 sine tapers. After regularization (57), the spectral matrix estimates were used to derive partial coherences. The partial coherence is a nonparametric measure of conditional independence between pairs of time series, and was used here to assess the relationship between the [EEG1, EEG2, Resp] channels inside each epoch. With *p* = 4 channels, we have Npcoh = 6 relationships between pairs of channels. Partial coherence estimates and *p* values (H0: partial coherence = 0) were derived for all pairs of channels, at each epoch. Results were adjusted for false discovery rate (FDR) across number of pairs and number of epochs in the experiment. This analysis framework produces a set of slowly evolving partial coherences between the [EEG1, EEG2, EMG, Resp] channels, for each experiment and each frequency band of interest, and produces a set of epoch-sized partial coherences for each pair of channels. However, we only report partial coherence results for [EEG1, EEG2, Resp], which have been effectively adjusted for EMG effect.

#### Evolving partial coherence during exploration.

We then investigated the association between exploration bouts and evolutionary partial coherences between channels derived from the analysis framework described in the previous section. We pooled all experiments together and regressed partial coherence estimates against lagged exploration bouts (±3 time bins around exploration bouts), using multiple-multivariate regression in R (v.3.6.1). This was done first on all types of exploration cues (novel objects, odor, food), then split by exploration cue type. The results of interest from the regression models are the estimated change from baseline in partial coherence values before/during/after exploration bouts, with the null hypothesis being that partial coherence values do not change around exploration bouts. This process was performed separately for each frequency band of interest.

#### Evolving partial coherence during sleep.

To investigate the evolutionary partial coherences between channels at the time of odor introduction during sleep, we applied the same strategy as for exploration, but now taking odor introduction as *time 0*, and evaluating ±3 time bins around this point. This was done across all odor types, but only for trials in which the animal remained in NREM sleep for at least 20 s after odor introduction. Using multiple-multivariate regression in R (v.4.3.2), we analyzed the data using two sets of multiple linear mixed-effect models.

#### Weighted connectivity graphs.

To obtain a baseline picture of direct associations between channels when the mouse was not exploring, we created weighted connectivity graphs. The nonstationary framework was applied as aforementioned to open arena trials, with no stimulus present. Only the data points associated with time-periods when the animal was not moving in the open arena (defined as “still,” using methods described in *Movement classification*) were retained. These points were then aggregated together, to produce a graphical model (Fig. 6*C* and Supplemental Fig. S6.1, *A* and *B*), partial coherence estimate between each channel. A line was drawn between any two channels if both *1*) partial correlation > 0.15 and *2*) *P* value for partial correlation being greater than 0 was <0.05. The thickness of each line represents the partial coherence estimate, and solid/dashed lines represent significant/not significant *P* values, respectively. This analysis was applied separately for the delta, theta, and alpha frequency bands.

#### Movement classification.

Movement in the open arena trials for each animal was detected using root mean squared (RMS) on EMG, across sliding time bins of 2 s (irrespective of frequency band of interest), when the absolute RMS exceeded a conservative animal-specific threshold (10–20% of absolute RMS, meaning that muscle activity had to be very low for the animal to be classed as still). The resulting movement detection estimates, which took values of either 0 or 1 for each time point, were cross-validated against video recordings.

#### Assessment of effect of movement on respiration measurements.

To estimate the effect of movement on respiration recordings, we applied a linear mixed-effect regression model, where EMG root mean squared is treated as a fixed effect, and individual animal is treated as a random effect [Sniff_rate ∼EMG_root_mean_squared + (1|Animal_ID)]. This effectively assesses the correlation between Resp rate and EMG RMS after adjusting for individual animal variation. The resulting correlation coefficient between Resp rate and EMG RMS is calculated from the effect size resulting from the regression model adjusted for standard deviations of Resp rate and EMG RMS. The resulting *P* value associated with this correlation coefficient is taken from the regression model. This regression analysis was repeated three times: *1*) on all time points, *2*) on time points associated with nonmoving moments (EMG RMS below animal-specific threshold), and *3*) on time points associated with moving moments (EMG RMS above animal-specific threshold).

#### Spectral power plots.

The preprocessed data were used for estimating the band power without regularization. Band power in the open arena experiment (including moving and nonmoving periods, *n* = 3 mice) was extracted from the spectral estimate matrix for each channel (i.e., diagonal entries of the spectral matrix) at each frequency over the range 0.5–20 Hz, and normalized against total average power over the same frequency range.

## RESULTS

### Implanting Thoracic Pressure Sensor in the Right Jugular Vein of Mice

To measure respiration in freely moving mice, we implanted 6- to 8-wk old mice with a wireless pressure sensor (Fig. 1, *A–F*). Briefly, after exposing the right jugular vein, we ligated the superior portion of it to stop any blood flow (Fig. 1*C*). Next, we made a small incision (Fig. 1*D*) through which we inserted the pressure sensor toward the thoracic cavity while monitoring the pressure signal online (Fig. 1*E*). Once we reached the optimal position of the probe, the sensor was held in place with an additional ligature around the vein (Fig. 1*F*). (Fig. 1; described in detail in materials and methods.)

### Comparing Thoracic Pressure Sensor and Flow Sensor Signals

**Figure 2.**
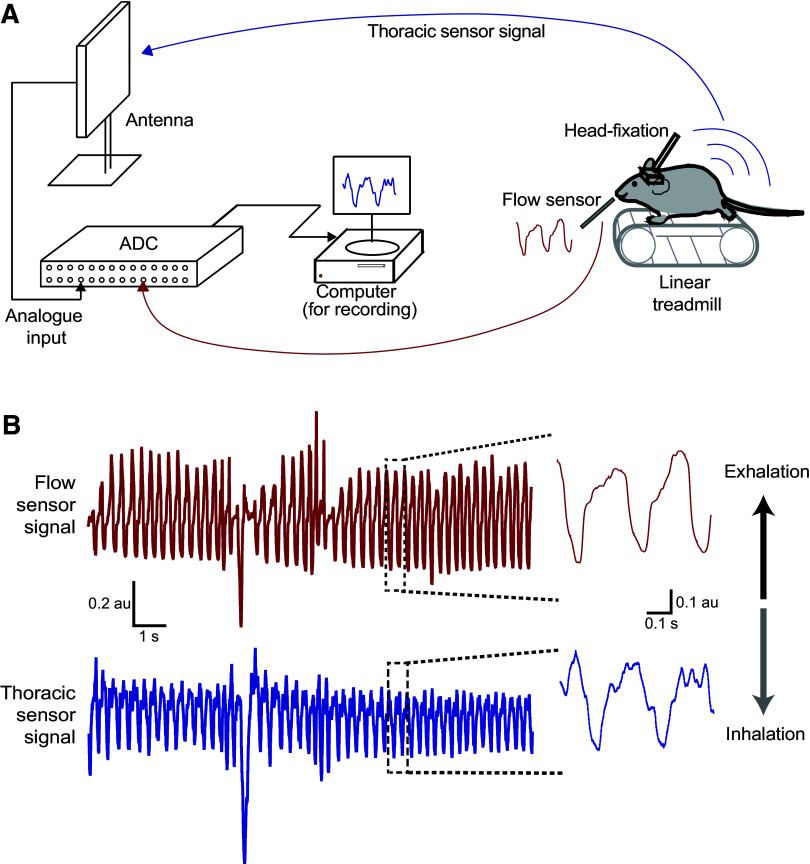
Head-fixed experimental setup for simultaneous measurement from nasal flow sensor and thoracic pressure sensor. *A*: a schematic diagram of the experimental setup used for simultaneous measurement of real-time respiration signals using a flow sensor placed near the nostrils and a thoracic sensor implanted in the jugular vein of head-fixed mice. *B*: an example recording obtained simultaneously from the flow sensor (red) and the thoracic sensor (blue). A zoomed section is shown at the right.

**Figure 3.**
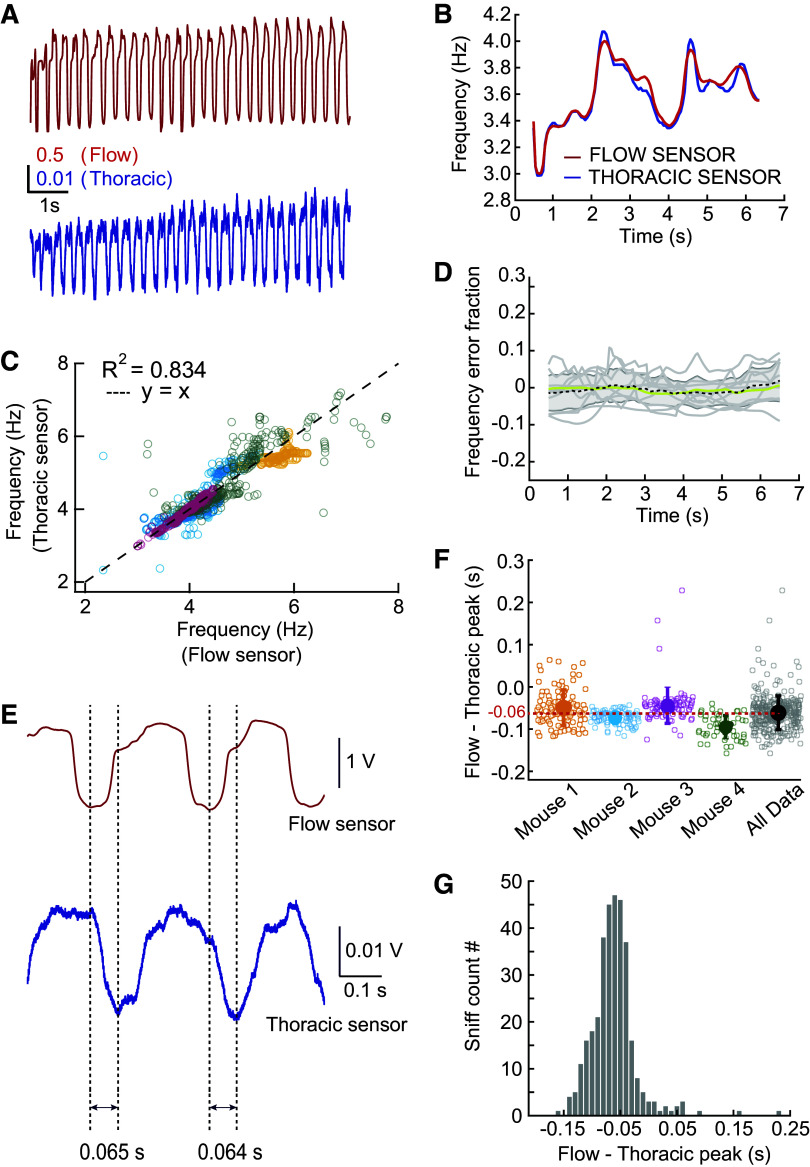
Implanted thoracic sensor is a reliable way of measuring respiration in awake mice. *A*: an example recording obtained simultaneously using a flow sensor (red) and a thoracic sensor (blue) in a head-fixed mouse. *B*: average frequency (estimated using autocorrelation with a rolling 1-s window, see materials and methods) from the two traces in *A*. *C*: frequency estimate using the thoracic sensor vs. flow sensor. Each marker represents the average frequency obtained from 1-s segment of a trial. (Linear Regression analysis, *R* = 0.914, *P* < 0.001). Different colors represent different animals. *D*: error fraction in estimating frequency from the same traces as in *C*. *E*: an example stretch of sniffs demonstrating the consistency of time-interval between the measured inhalation peak using the two sensors. *F*: time-interval between the inhalation peak times measured from the two sensors simultaneously. The colors represent the same animals as in *C*. Solid markers represent means ± SD. The black marker represents the population average time-interval of −0.06 ± 0.03 s. *G*: histogram of the population of time difference in inhalation peak obtained from all the sniffs in *F*, *n* = 350.

After recovering from the surgical implantation (>7 days after surgery), mice were transferred onto a treadmill. After a short habituation session on the treadmill, we recorded the change in thoracic pressure signal using the implanted pressure sensor, and the change in nasal flow using a flow sensor placed in front of the nostrils (Fig. 2*A*). Both the sensors reflect respiration in the animals. Qualitatively, the signals looked similar, with downward deflection representing inhalation in both recording methods (Fig. 2*B*, Supplemental Fig. S2.1). To quantitatively compare these methods, we measured the frequency and peak inhalation time in both signals recorded simultaneously (Fig. 3). Using autocorrelation to estimate the average frequency, we did not observe any significant difference in the estimated frequency (*R* = 0.914; linear regression) between the two signals (Fig. 3, *A–C*) and the relative error between respiration frequency measurements derived from thoracic pressure and flow sensor measurements was 0.016 ± 0.009 (means ± SD; *n* = 21 traces; 4 mice, Fig. 3*D*).

As nasal flow and thoracic pressure reflect different aspects of respiration, there is likely to be a consistent shift in the time at which each signal peaks and knowing this shift could enable “translation” between these signals. We observed that the end of inhalation corresponds to the point of minimum thoracic pressure, and that the peak of inhalation preceded this point by 0.06 ± 0.03 s (*n* = 350 sniffs; 4 mice). Overall, therefore, we have found that the thoracic pressure signal can be reliably used to measure respiration and to detect the time of peak flow inhalation. Although we assume that this linear relationship holds for the higher frequencies observed during active exploration (Fig. 5), it is an important caveat that we could only experimentally describe this relationship for the frequencies naturally demonstrated under head-fixation conditions (∼2–8 Hz). Interestingly, Reisert et al. (46) were able to describe a similar relationship between thoracic pressure and intranasal pressure across a wider range of sniffing frequencies, and found that the variability in the difference between these two pressure signals was smaller at higher frequencies (their Figure 5).

### Freely Moving Mice Have a Bimodal Respiration Frequency Not Associated with Running Speed

**Figure 4.**
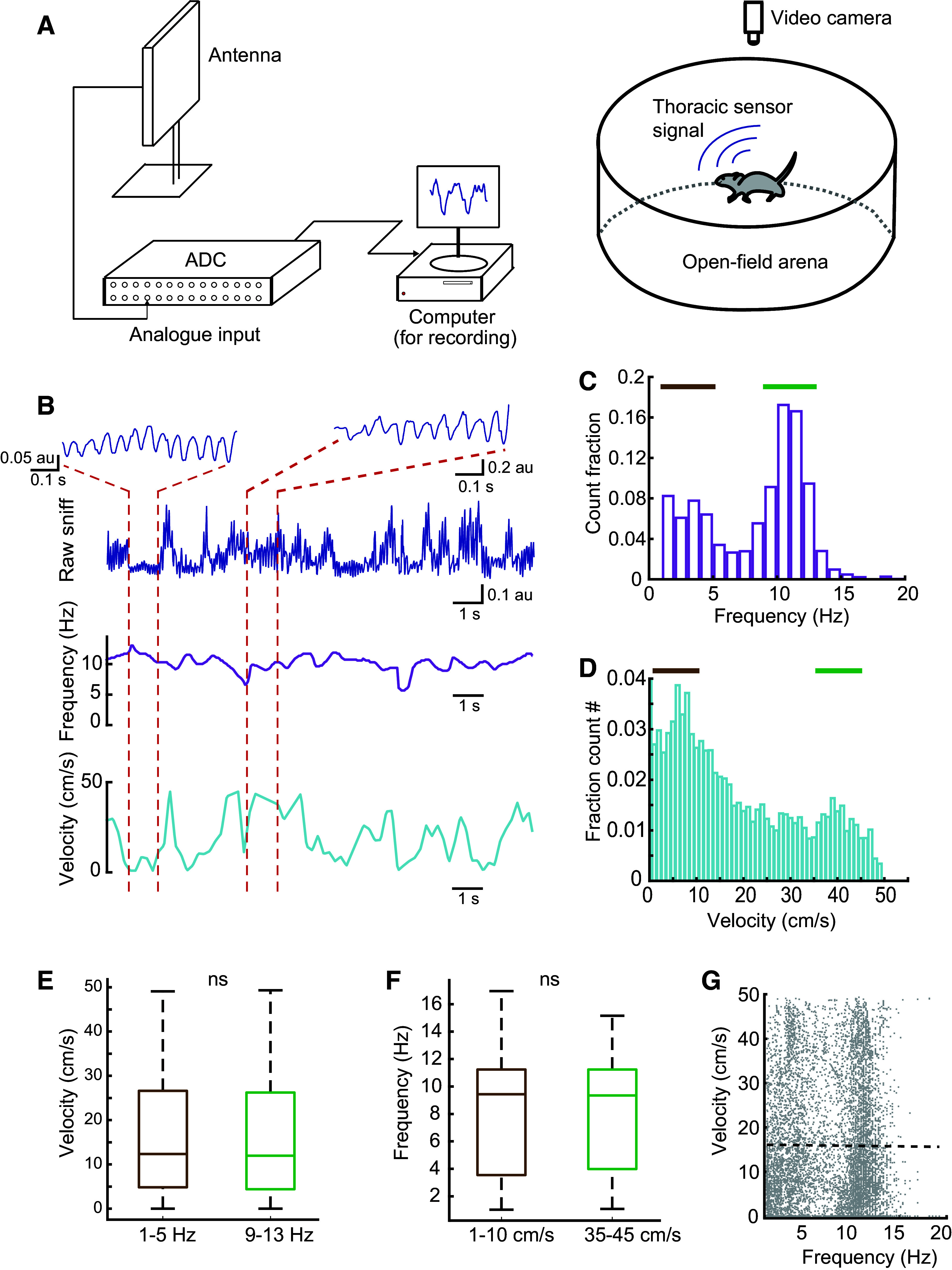
Respiration measurement from freely moving mice implanted with thoracic sensors exploring an open arena. *A*: a schema of the experimental setup used for telemetry recording of respiration in freely moving animals in an open arena. *B*: an example trace of respiration recording from an animal while it freely navigated in the open arena (*top*). Note the respiration profiles in the two zoomed sections during high frequency respiration, low velocity movement (*left*), and high frequency, high velocity (*right*). *C*: histogram of respiration frequency estimate from all animals (*n* = 4 animals). Brown and green indicate the range of frequencies analyzed in *E*. *D*: histogram of running velocity from all animals in *C*. Brown and green indicate the range of velocities analyzed in *F*. *E*: average running velocity was independent from respiration frequency in frequency ranges between 1 and 5 Hz (brown) and 9 and 13 Hz (green, indicated in *C*). *F*: average respiration frequency was independent from running velocity at speeds of 1–10 cm/s (brown) and 35–45 cm/s (green, indicated in *D*). *G*: running velocity vs. respiration frequency does not show any substantial correlation (*R* = −0.0071, dotted line) from all the animals in *C*.

Next, we used this method to measure respiration in freely moving mice while they explored an open arena. The thoracic pressure recording was acquired after a short period (∼2–3 min) of habituation in a novel arena while movements were video-recorded for postanalysis (Fig. 4, *A* and *B*). The population of respiration recordings from all animals revealed a bimodal distribution of respiration frequency (Fig. 4*C*) with a small peak at approximately 3 Hz and a larger peak around 11 Hz. (Note that a bimodal distribution was also observed by Reisert et al. (46), but in their study it was the lower frequencies that were more common. We suggest that this difference may come from the recording arenas—while the Reisert et al.’s mice were in their home cage, our mice were in a large, novel arena, which may have promoted more exploratory sniffing behavior in the higher frequency range.) The estimated velocity from simultaneously acquired video recordings also showed a bimodal distribution (Fig. 4*D*) with peaks at approximately 7 cm/s and 40 cm/s. To understand whether respiration frequency was linked to running speed, we quantified the average velocity associated with two prominent frequency bands (1–5 Hz and 9–13 Hz) (Fig. 4*E*) and the average frequency associated with the two prominent bands of velocity (1–10 cm/s and 35–45 cm/s) (Fig. 4*F*). Surprisingly, we did not observe any significant difference in either of the cases (velocity comparison: *P* = 0.4691; and frequency comparison: *P* = 0.2501; both unpaired *t* tests), suggesting that respiration rate was largely independent of running velocity (correlation coefficient = −0.0071, Fig. 4*G*). These results suggest that respiration rate is not simply linked to motor activity, and fits with previous work showing similar differences in respiration rhythm (peaks at 3–5 Hz or 9–11 Hz) during head-fixed running on a treadmill (32). Interestingly, recent work has shown that respiration changes are closely linked to subtler behavioral features during exploration, such as rearing and foraging (49).

### Exploration of Environmental Cues Significantly Increases Respiration Frequency

**Figure 5.**
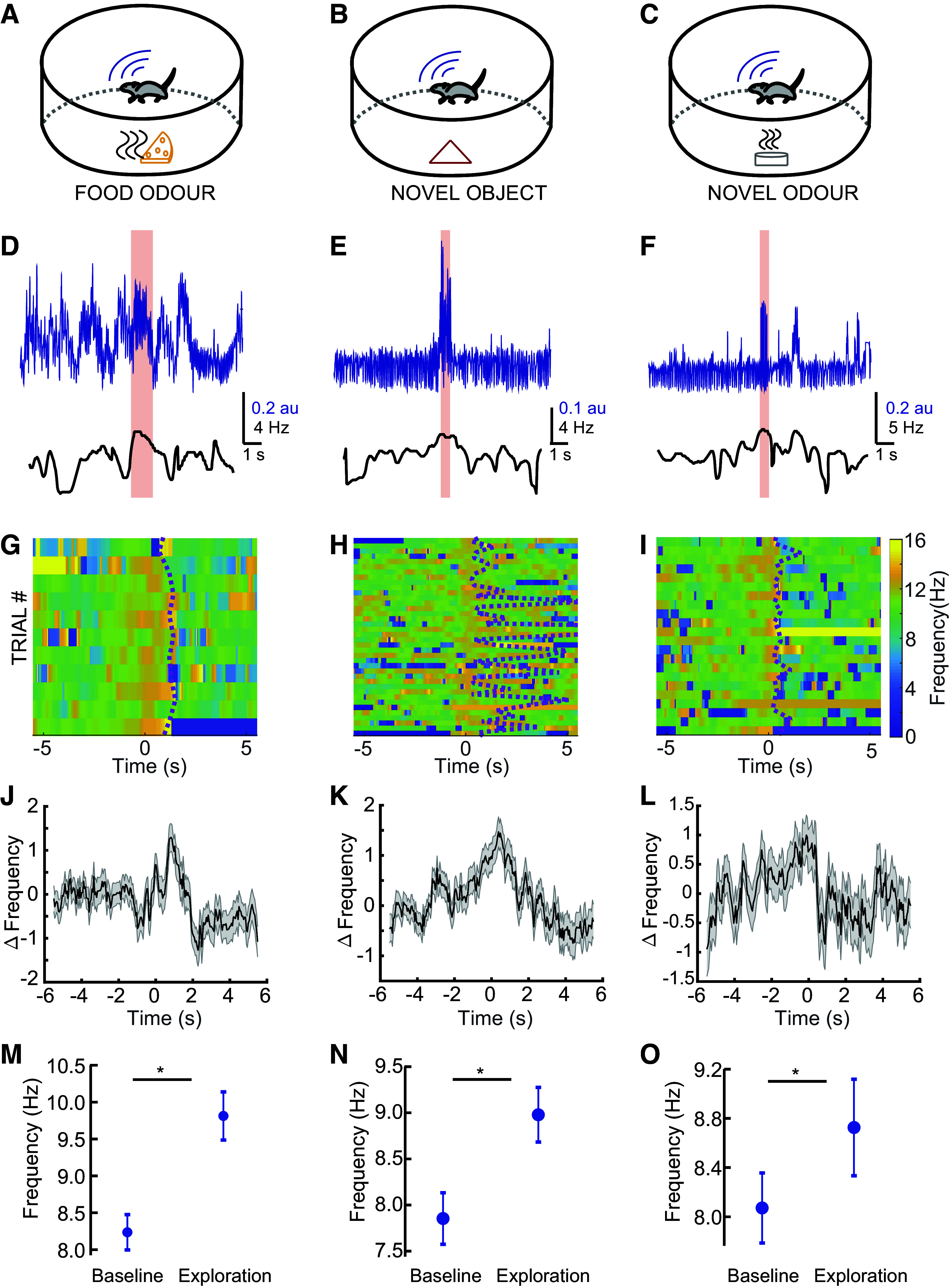
Mice show increased respiration frequency during exploring novel food, object, and odor. A schematic diagram of the experimental setup used for mice exploring food (*A*), a novel object (*B*), and a novel odor while recording their respiration (*C*). *D*: an example respiration recording (*top*) and its corresponding respiration frequency estimate (*bottom*) for a trial where an inaccessible piece of food was placed in the arena. The pink bar denotes the time of exploration in that trial. Similar example for a trial in which a novel object (*E*) and novel odor (*F*) were placed in the arena. Respiration frequency plots from multiple trials performed by an animal exploring the inaccessible food (*G*), novel object (*H*), and novel odor (*I*). Note the change in sniff frequency during the exploration time. Dotted line indicates the time at which the animal stopped exploring for each trial. Baseline subtracted respiration frequency plotted against time for inaccessible food (*n* = 85) (*J*), novel object (*n* = 134) (*K*), and novel odor (*n* = 72) (*L*). The thick lines represent the mean and the shaded region represents the SE (4 mice). Respiration frequency during the period of exploration significantly increases from the baseline respiration frequency for inaccessible food (*P* = 0.0014) (*M*), novel object (*P* < 0.001) (*N*), and novel odor (*P* = 0.03) (*O*) (4 mice). *Significant difference (*P* < 0.05).

We next asked how respiration frequency changes when mice encounter cues such as novel objects (e.g., rubber duck, plastic toys, empty bottles), monomolecular chemical odorants (ethyl butyrate, 2-hexanone, eucalyptol, and amyl acetate), and food (inaccessible pellet). After habituation to an empty arena, we placed a cue from each class into the arena, one at a time in a random order (Fig. 5, *A–C*). We monitored respiration as the mice voluntarily explored these different cues (Fig. 5, *D–F*). We then calculated the respiration frequency across all exploration bouts (Fig. 5, *G–I*) and quantified the change in respiration frequency from the baseline after aligning the trials to the initial exploration video frame (Fig. 5, *J*–*L*). For all the three classes of cues, we observed that the mice increased their respiration frequency significantly (food odor: *P* = 0.0103; novel object: *P* = 2.6*e*-5; and novel odor: *P* = 0.0394; paired *t* tests) at the moment of stimulus investigation, even against the relatively high baseline frequency that mice displayed in this exploratory state (Fig. 5, *M*–*O*). Sniffing rates are well known to increase during tasks that rely on olfactory discrimination (4, 6, 7, 40, 58–60), but the present results, along with other results showing increased sniffing during exploratory locomotion even in the absence of any cues (26), suggest that raised respiration may be a general feature of exploration. In turn, neural entrainment to this respiration rhythm may act similarly—but distinctly—to theta oscillations, providing a scaffold for long-range network communication across the brain (32). We therefore sought to examine how the respiration changes during voluntary exploration are related to brain-wide activity in defined frequency bands.

### Exploration-Triggered Respiration Changes Increase or Decrease the Coupling between Respiration and Brain Activity Depending on EEG Frequency Band

Recent results have shown that cortical dynamics can be altered by changes in respiration, which were triggered experimentally by changes in CO_2_ levels (28). We sought to test how the sniffing changes during voluntary exploration related to cortical dynamics.

**Figure 6.**
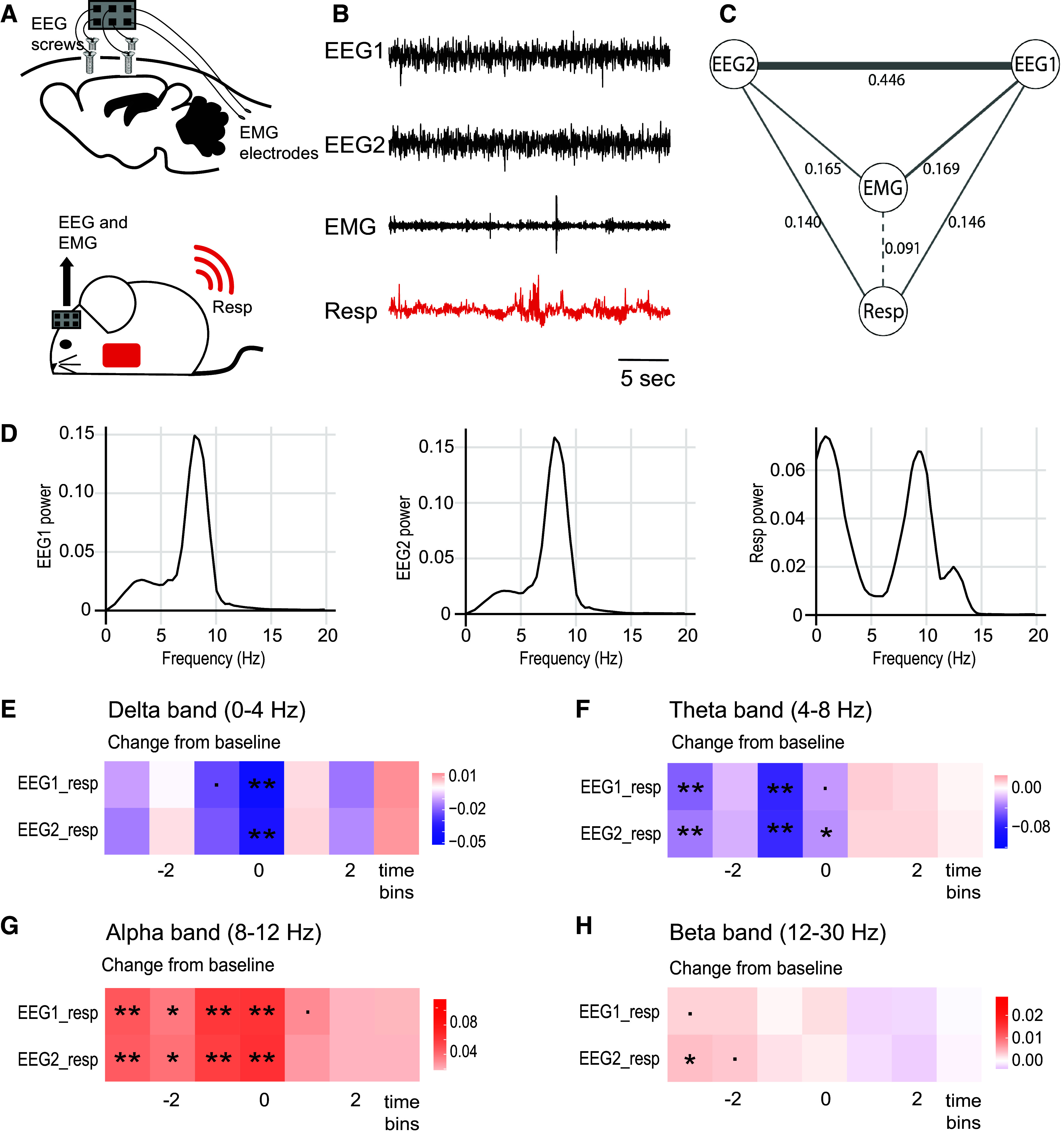
EEG, electromyogram (EMG), and sniff relationships during freely moving awake behavior. *A*: schematic of EEG/EMG electrode placement (*top*) and mouse implanted with both thoracic sensor and EEG/EMG recording devices (*bottom*). *B*: example raw traces from each of the four recording channels. *C*: graphical representation of the baseline relationships between the four recording channels. Partial coherence between each channel was estimated while the animals were awake but not moving in an open arena, in the absence of additional environmental cues. A line was drawn between any two channels if both *1*) partial correlation > 0.15 and *2*) *P* value for partial correlation being greater than 0 was <0.05. The thickness of each line represents the partial coherence estimate, and solid/dashed lines represent significant/not significant *P* values, respectively. This plot is for the theta band (see Supplemental Fig. S6.1 for other bands). *D*: spectral power plots for EEG and Respiration (Resp) channels recorded in the open arena (including moving and not moving epochs), showing brain activity and breathing signals in the delta-beta frequency ranges (*n* = 3 mice). *E*–*H*: estimates of changes in partial coherence between channel pairs in time bins before (negative time lag), during (0 time lag), and after (positive time lag) an animal explores an introduced environmental cue (either novel object, novel odor, or food odor). Analysis was done separately for different frequency bands: delta (*E*; 4 mice), theta (*F*; 4 mice), alpha (*G*; 4 mice), and beta (*H*; 4 mice). Key for significance: **P < 0.01; *P < 0.05; ·*P* < 0.1.

Using the implanted thoracic pressure sensor in combination with simultaneous EEG/EMG recording (Fig. 6, *A* and *B*), we first explored how animals’ respiration is related to brain activity while the animal was not exploring or engaged in any observable motor activity (“still” in the open arena, classified according to EMG measurement, see materials and methods). We applied a frequency-domain locally stationary time series analysis framework (see materials and methods) under the assumption of stationarity. This allowed us to build a baseline picture of the relation between all four recording channels (2 × EEG, 1 × EMG, 1 × Resp). We found that respiration was not significantly related to the EMG channel, but was directly related to the two EEG channels (which were also directly related to each other, and to EMG; Fig. 6*C* shows this analysis for the theta band, Supplemental Fig. S6.1, *A* and *B* shows the same analysis for delta and alpha bands).

We then used a frequency-domain approach called partial coherence to understand how the links between these channels evolve over a period of exploration. Partial coherence is a nonparametric measure of direct relationships between signals in a multivariate set. It is derived from the spectral density of the entire channel system, at any given frequency. It is valued between 0 and 1, and can be interpreted as the fraction of power shared by any two channels (in our case, EEG and Resp, power spectra across animals shown in Fig. 6*D*), while controlling for the influence of other channel (EMG) (57; and see materials and methods for more details).

For four frequency domains: *1*) delta band, 0–4 Hz; *2*) theta band, 4–8 Hz; *3*) alpha band, 8–12 Hz; *4*) beta band, 12–30 Hz, we examined how the association between EEG and Resp channels changed before, during, and after the mice explored environmental cues (while controlling for the influence of muscle activity recorded in the EMG channel), using a slowly evolving locally stationary (SeLV) framework. We found that, for the delta and theta frequency bands, the partial coherence across channels decreased relative to baseline before and during an exploration bout, increasing again after the animal stopped exploring (Fig. 6, *E* and *F*). In contrast, for the alpha and beta frequency bands, the partial coherence across channels increased relative to baseline before and during exploration, decreasing again after the animal stopped exploring (significantly so for alpha but not beta; Fig. 6, *G* and *H*). This suggests that there is a direct relationship between respiration and brain activity, not mediated by muscle movement, which fluctuates significantly during exploration bouts. This change in direct signaling between brain and respiration differed based on frequency band: The relationship between respiration and brain activity in the delta and theta range decreased before and during exploration, whereas for the higher frequency bands—alpha and beta—the relationship between brain activity and respiration increased before and during exploration. For all frequency bands, these trends were independent of cue class (novel object/chemical odorant/inaccessible food).

Interestingly, Martin et al. (61) found increased coherence in the beta range between the olfactory bulb and hippocampus during an olfactory learning task, and Zhong et al. (26) found that exploration was associated with increased alignment between respiration rhythm and fast gamma oscillations within the olfactory bulb and the prelimbic cortex. Our results suggest that behavior-dependent association between neural oscillations and respiration-driven rhythms could be a widespread pattern across cortical as well as subcortical areas. They also extend the picture across frequency domains, and it is interesting to note that the decreased relationships between respiration and brain activity occur in frequencies normally associated with rest or sleep (delta and theta), whereas increased association between respiration and brain activity are found within frequencies normally associated with arousal, sensory awareness, and attention (alpha and beta).

### Respiration Patterns Vary across Vigilance States

**Figure 7.**
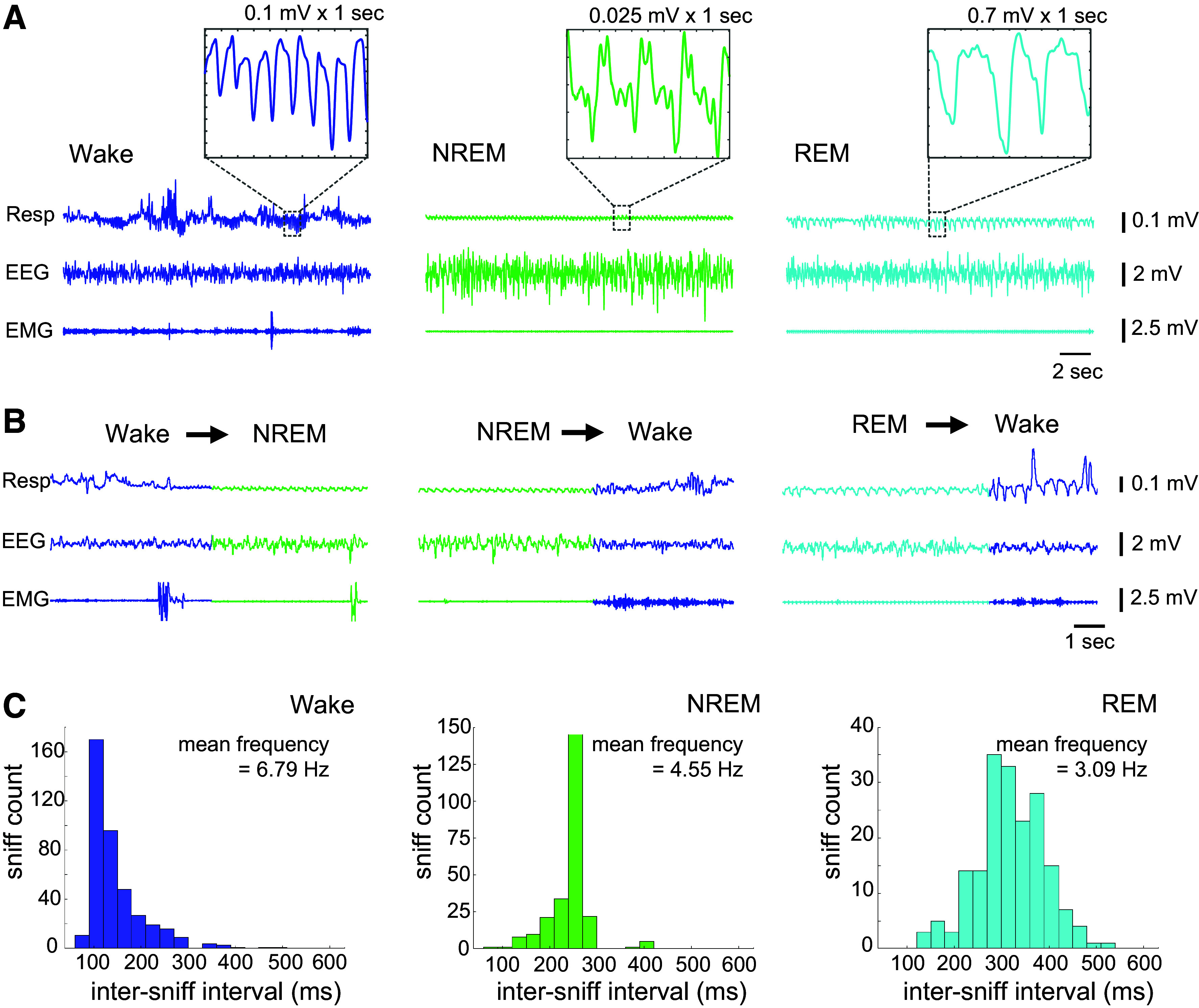
Respiration frequency changes with vigilance state. *A*: raw example sniff, EEG, and electromyogram (EMG) signals in wake (blue), non-rapid eye movement (NREM, green), and rapid eye movement (REM, cyan) sleep. The respiration trace (*top row*) shows that respiration is varied and often large in amplitude during wake, highly regular and low in amplitude during NREM sleep, and slightly irregular during REM sleep. Respiration frequency tends to be high during wake and lower in NREM and REM sleep (zoomed in boxes). *B*: changes in respiration are seen almost instantaneously upon transition between different vigilance states (note, mammals typically do not transition from REM to NREM sleep). *C*: distributions of intersniff intervals for wake, NREM, and REM sleep, with mean respiration frequency for each state given in Hz.

We next examined how respiration varied as the animal cycled through periods of wake and different sleep stages in its home cage. Similar to previous work using different methods of respiration recording [e.g., implanted thermocouples (26); whole body plethysmograph (28)], we found that respiration patterns were different between different sleep states (Fig. 7*A*), tending to be high in frequency and amplitude but highly variable during wake; very regular and low in frequency and amplitude during NREM sleep, and slightly irregular in both frequency and amplitude during REM sleep. Switches between these different respiration signatures occurred almost instantaneously upon transition between different vigilance states (Fig. 7*B*). The distribution of intersniff intervals was different between sleep states, leading to a mean respiration frequency that was highest in wake (mean frequency = 6.79 Hz) and lowest in REM sleep (mean frequency = 3.09 Hz) (Fig. 7*C*).

### Odor-Triggered Respiration Changes during NREM Sleep Do Not Induce Changes in Sleep-Associated Brain Rhythms

**Figure 8.**
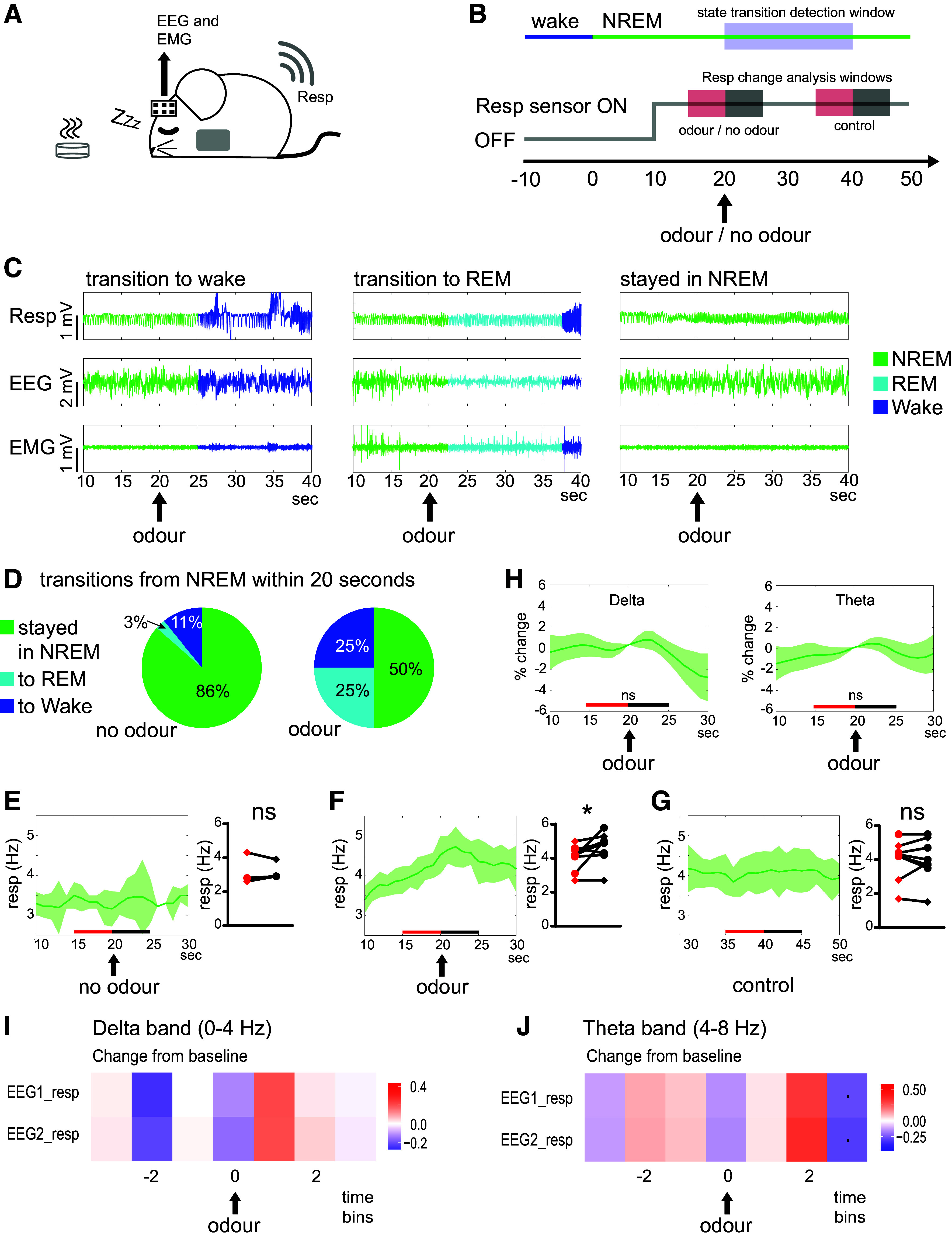
Odor presentation during sleep. *A*: schematic of recording setup. While the animal was sleeping, EEG/EMG and respiration signals were recorded, and odors were placed in the animal’s home cage. *B*: schematic of analysis framework. Ten seconds after the animal entered NREM sleep (switch from blue to green, *top row*), the respiration sensor was turned on (for 1 min; gray line, *middle row*). Ten seconds later, odor was placed into the cage and the window for detecting state transitions was opened (for 20 s, blue shaded square). The red and black shaded squares represent the timing of respiration frequency comparisons (*E*–*G*) and EEG frequency band comparisons (*H*). *C*: raw example traces of respiration (Resp), EEG, and EMG signals for an instance when the animal transitioned to wake, REM, or NREM, within 20 s of odor presentation. *D*: pie charts showing proportion of state transitions that occurred between 20 and 40 s from the onset of NREM sleep, for cases where odor was not presented (*left pie chart*) and cases where odor was presented at *t* = 20 s (*right pie chart*). While odor presentation did increase the proportion of transitions to wake and REM sleep, in half the cases, odor presentation did not cause the mouse to switch out of NREM sleep (10 out of 20 trials, across two mice). *E*: in three trials, the respiration sensor was turned on without any odor being presented. In these cases, respiration frequency did not change 20 s after the start of NREM sleep (*left*: dark green line represents mean across trials, shaded region represents SE; *right*: paired *t* test, *P* = 0.16, two shapes represent two mice). *F*: in the cases where odor presentation did not cause transition out of NREM sleep, there was a significant increase in the average respiration frequency (paired *t* test, *P* = 0.021; colors and shapes as in *E*). *G*: control comparisons made 20 s after odor presentation (in cases where animal was still in NREM sleep) did not reveal a change in average respiration frequency (paired *t* test, *P* = 0.79; colors and shapes as in *E*). *H*: in cases where odor presentation did not cause transition out of NREM sleep, there was no significant change in the EEG delta power (*H*; *P* = 0.40) or theta power (*I*; *P* = 0.31) (colors as in *E*–*G*; *P* values are from paired *t* tests, comparing average values for 5 s shown in red vs. 5 s shown in black, as in *E*–*G*). *I* and *J*: estimates of changes in partial coherence between channel pairs in time bins before (negative time lag), during (0 time lag), and after (positive time lag) an odor was introduced to the animal during NREM sleep (as for *F*–*H*, only including trials in which the animal remained in NREM sleep, 10 trials across two mice). Analysis was done separately for the dominant sleep frequency bands: delta and theta. Key for significance: *P < 0.05; ·*P* < 0.1.

Girin et al. (28) found that the respiration changes induced by alterations in CO_2_-enriched air were capable of driving brain activity changes during wakefulness but not during sleep. We wanted to explore whether stimulus-induced sniff changes might tell a different story during sleep. We allowed the animal to rest in its home cage while monitoring EEG and EMG signals continuously (Fig. 8*A*). When the animal entered NREM sleep, we turned on the thoracic pressure sensor (it was not on continuously due to battery constraints—see materials and methods for details), and subsequently presented an odor to the sleeping animal (Fig. 8*B*). Within the 20 s following odor presentation, animals transitioned to wake 25% of the time, to REM sleep 25% of the time, and stayed in NREM sleep 50% of the time (Fig. 8, *C* and *D*). When no odor was presented, the animal was more likely to stay in NREM sleep, and respiration did not change (Fig. 8, *D* and *E* paired *t* test, *P* = 0.16). But in the cases where odor presentation did not trigger a transition out of NREM sleep, we were able to monitor the effects on respiration and brain activity. In such cases, respiration increased (Fig. 8*F* paired *t* test, *P* = 0.021; cf no change 20 s later: Fig. 8*G* paired *t* test, *P* = 0.79), but no significant change was observed in sleep-related EEG rhythms [Fig. 8*H* delta: *P* = 0.40; theta: *P* = 0.31; theta:delta ratio: *P* = 0.91 (not-depicted); paired *t* tests]. In line with these results (and in opposition to the awake exploration results), we found that directly examining the partial coherence between respiration and brain activity during sleep (exactly as applied during awake exploration, Fig. 6, *E–H*), did not reveal a change at the time of odor introduction (Fig. 8, *I* and *J*). Thus, our sleep results align with Girin et al.’s findings: neither CO_2_-induced nor stimulus-induced sniff changes appear to drive brain activity changes during sleep.

## DISCUSSION

We present a new implantation technique for a wireless thoracic pressure sensor, used to monitor respiration in conjunction with brain recording in freely moving mice. After calibrating with head-fixed respiration measurements using flow sensors, we were able to use this method to investigate respiration and its relationship to EEG brain activity in a variety of behavioral contexts: free exploration of novel environments and cues, and across different sleep-wake states.

Although changes in respiration in response to olfactory discrimination odor cues have been well documented (4, 40, 58–60), our data reveal comparable sniffing increases across voluntary exploration episodes of novel odors, novel objects, and inaccessible food. It is worth noting here that the “odor” stimuli are not the only stimuli that have an olfactory component. Food pellets of course have an olfactory component that is both familiar and holds intrinsic value, whereas the novel objects are each likely to have different odors that may vary in their strength and complexity depending on the type of material. We expect that the mice are using a multimodal (primarily olfactory and visual) approach to explore each class of stimuli, and while the specific neural response within olfactory and visual regions is likely to depend on the relative salience of each cue, our respiration data align with long-standing observations that diverse sensory stimuli (including nonolfactory stimuli) can arouse sniffing (9), suggesting that raised respiration is a general behavioral feature of active exploration in mice. However, there may be subtler links between respiration and behavior that we have not been able to draw out from the current data. For instance, Liao and Kleinfeld (49) recently showed that the relative phase between head movement and breathing is different depending on whether an exploring animal is rearing or foraging. It would be interesting to repeat our exploration experiment with the addition of head and torso sensors or higher frame-rate videos from multiple angles in combination with unsupervised machine learning analysis (62, 63), to examine whether there are also differences in the respiration-head movement relationship according to whether the animal is exploring objects, odors, or food. Such data could help reveal whether this relationship varies continuously across behavioral states, or whether there is something special about respiration during the specific state of foraging, or in cases of novelty versus familiarity.

Given the increasing view that respiration may provide the scaffold for a brain-wide rhythm that can help coordinate neural information transfer across brain regions (22–25, 29), we sought to understand how exploratory sniffing in a voluntary, freely moving task was related to cortical activity in specific frequency bands. Using cortical EEG recording we found that, during stimulus approach, there was a decrease in the association between sniffing and EEG activity in both the delta and theta frequency bands. Cortical delta frequency is most prominent during deep sleep, whereas cortical theta frequency is associated with REM sleep (reviewed in Ref. 64), and so it is perhaps intuitive that the respiratory link with these rhythms would be reduced as the mouse makes an active exploratory approach. The partial coherence between respiration and EEG in the alpha band, on the other hand, increases during exploration. Cortical alpha rhythms have long been associated with conscious perception (reviewed in Ref. 65), and in particular, the likelihood of awareness has been found to vary with the phase of the alpha rhythm in humans (66–70). It may therefore be of great functional benefit to align sniffing with this rhythm when an animal actively examines a new object. We observed a slight (but not significant) increase in the partial coherence between respiration and brain activity in the beta band, which is associated with motor preparation, sensorimotor integration, and top-down signaling (comprehensively reviewed in Ref. 65). In general, our data show that active exploration of sensory stimuli in a voluntary, freely moving setting trigger sniff changes that show transiently increased association with brain activity in specific frequency bands.

Several recent studies have revealed that neuronal entrainment of different brain rhythms to the respiration rhythm is affected by arousal (26–28, 30, 31), so we next looked at respiration across different sleep and wake states. We found that sniff amplitude, frequency, and variability are different between wakefulness, NREM sleep, and REM sleep, and that transitions between these vigilance states trigger instantaneous changes in sniff pattern. These results show that changes in brain state are linked to observable changes in respiratory behavior, even in the absence of any change in the olfactory environment, and are in line with what has previously been reported in human sleep (71, 72).

Interestingly, odor introduction during deep sleep is less likely than other sensory stimuli to produce EEG signs of arousal in humans (73), and may even deepen sleep (74), although these studies did not examine respiratory changes. Here, we found that introducing an odor to the animal’s home cage during NREM sleep could trigger an increase in sniffing in the absence of any vigilance state changes, and without significantly altering the partial coherence between respiration and the dominant sleep brain rhythms. This suggests that, during sleep, active sampling can be modulated even without any obvious changes to the overall arousal state of the brain, and that the association between respiration and brain activity is not as easily altered as during wake. Our results also expand on those of Girin et al. (28), who showed that breathing changes triggered not by an olfactory stimulus but by changes in CO_2_ concentration, were least capable of driving respiration-related brain activity changes during deep sleep.

Interestingly, in humans it has been found that presentation of a task-associated odor during sleep can improve learning, both in laboratory (75) and real-world (76, 77) settings. This cognitive improvement is thought to be the result of targeted memory reactivation (TMR), whereby the content and frequency of hippocampal replay events during sleep are biased by a sensory stimulus (shown with auditory stimuli: see Ref. 78). Because such replay events would not be detected by the EEG measurements made here, it is possible that the odor presentation during sleep is causing a change to brain activity that we are missing. An additional factor is the salience of the sensory cue presented during sleep—our odors were neither novel (having been presented once during wake) nor associated with any particular task or outcome. It would be interesting to repeat this experiment with task-associated odors and sleep-novel odors, to see whether stimulus salience affects the sniffing response, the EEG response, or their coherence in a particular frequency band.

We would like to point out some limitations of the present work. One major drawback of this wireless technique to measure respiration is that the current version of the implanted device for mice cannot be recharged, so the absolute data collection period is limited to approximately 24 h. For this reason, the experimenter must make a choice between monitoring in long stretches over 1–2 days or in short bursts over several days/conditions. We chose the latter approach, with each respiration recording lasting between 30 s and 2 min. This ensured that we were able to do many recordings across different exploratory and vigilance conditions, but of course a different approach would need to be adopted to examine behaviors that evolve over a slower timescale, in which case the number of experimental conditions that an individual mouse could participate in would be smaller. However, this constraint would be less harsh for the rat device, which can house a bigger battery, and may be further reduced in both species with the rise of wireless charging. Improved batteries/charging might also provide the possibility of implanting smaller thoracic pressure sensors in younger animals to allow for developmental studies.

Another potential issue with any new recording technique is the possibility for signal contamination by unexpected confounds. The confound that we were most concerned with here was movement: if the pressure sensor was affected by locomotion or head movements, for instance, then we could not be sure whether changes in the signal were the result of changes in breathing or in movement. Similarly, we could not meaningfully compare across conditions that differ in movement levels, as is the case for wake and sleep. We therefore carried out multiple tests to examine whether muscle movement was contaminating the respiration signal picked up by the pressure sensor. First, we found that respiration measurements were not correlated with either the speed of mouse locomotion (as measured by video-tracking, Fig. 4, *E–G*), or tone of the neck muscle (as measured by EMG, Supplemental Fig. S6.1*E*). Second, we found that estimates for evolving partial coherence between EEG and respiration did not differ when the mouse was still versus moving (as defined by EMG, Supplemental Fig. S6.1, *C* and *D*). And finally, we examined the weights of the direct associations calculated using stationary coherence, and found that the link between respiration and EMG was the smallest across frequency bands (Fig. 6*C*; Supplemental Fig. S6.1, *A* and *B*). We are therefore relatively confident in this case that movement artifacts did not impact the respiration measurements made by the implanted pressure sensor. We cannot rule out that the sensor might pick up movement to some degree, but if this was the case here, the respiration signal was certainly strong enough not to be affected by it.

In summary, we have used a new surgical technique to implant a telemetry-based thoracic pressure sensor, and have shown that this can be used to accurately measure respiration in freely moving mice. Since respiration rhythm is increasingly being viewed as an important brain-wide scaffold to which other neural rhythms can align, we combined this new measurement technique with implanted EEG and EMG to monitor brain activity in specific frequency bands during behavior and across vigilance states. During stimulus exploration, the association between respiration and cortical delta and theta decreased, but the association between respiration with alpha increased. Odor presentation during sleep was able to cause a transient increase in sniffing, but did not appear to change dominant sleep rhythms in the cortex. Overall, our data align with the idea that respiration may be a useful driver for synchronizing specific brain rhythms, particularly during wakefulness and exploration, but during sleep, respiratory changes seem less able to impact EEG measures of brain activity.

Having demonstrated the flexibility with which thoracic pressure sensing can be combined with different behavioral assays and brain recording, we anticipate that this wireless technique to measure respiration will provide many new insights into the way that animals use olfactory information to understand the environment. Our recent findings show that mice can compute subsniff level information (41, 43), and it will next be important to interrogate how the relationship between respiration and specific brain rhythms contributes to subsniff level computation in freely moving animals.

## DATA AVAILABILITY

The data that support the findings of this study are available from the corresponding author, upon reasonable request.

## SUPPLEMENTAL DATA

10.6084/m9.figshare.25624752 Supplemental Figs. S2.1 and S6.1: https://doi.org/10.6084/m9.figshare.25624752.

## GRANTS

This work was supported by the Francis Crick Institute which receives its core funding from Cancer Research UK under Grant CC2036, the UK Medical Research Council under Grant CC2036, and the Wellcome Trust under Grant CC2036; by the UK Medical Research Council under Grant Reference MC_UP_1202/5; a Wellcome Trust Investigator Grant 110174/Z/15/Z (to A.T.S.) and the National Science Foundation/Canadian Institute of Health Research/German Research Foundation/Fonds de Recherche du Québec/UK Research and Innovation–Medical Research Council Next Generation Networks for Neuroscience Program under Award No. 2014217.

## DISCLOSURES

No conflicts of interest, financial or otherwise, are declared by the authors.

## AUTHOR CONTRIBUTIONS

D.D., A.T.S., and J.J.H. conceived and designed research; D.D. and J.J.H. performed experiments; D.D., D.S.-L., and J.J.H. analyzed data; D.D., A.T.S., and J.J.H. interpreted results of experiments; D.D., D.S.-L., and J.J.H. prepared figures; D.D., D.S.-L., A.T.S., and J.J.H. drafted manuscript; D.D., D.S.-L., A.T.S., and J.J.H. edited and revised manuscript; D.D., D.S.-L., A.T.S., and J.J.H. approved final version of manuscript.
